# Growth Hormone/Insulin-Like Growth Factor-1 Axis as Related to Body Mass Index in Patients with Idiopathic Short Stature

**DOI:** 10.4274/Jcrpe.901

**Published:** 2013-03-21

**Authors:** Pınar Cengiz, Firdevs Baş, Fatmahan Atalar, Ahmet Uçar, Feyza Darendeliler, Gökçe Akan, Tuğba Tarhan, Rüveyde Bundak

**Affiliations:** 1 İstanbul University Istanbul Faculty of Medicine, Department of Pediatric Endocrinology, İstanbul, Turkey

**Keywords:** Idiopathic short stature, insulin-like growth factor 1 (IGF-1), body mass index, IGF-1 receptor, IGF-binding protein 3 (IGFBP)-3, IGF-1 generation test

## Abstract

**Objective:** Idiopathic short stature (ISS) is a heterogeneous disorder. An impairment of growth hormone (GH)/insulin-like growth factor 1 (IGF-1)/IGF-1 receptor (IGF-1 R) axis is postulated. To evaluate the somatotropic axis in relation to body mass index (BMI), serum IGF-1, IGF-binding protein-3 (IGFBP-3) and the expression of IGF-1 R genes in patients with ISS.

**Methods:** Fifty-five ISS patients (24F/31M) aged 14.6±5.5 years (range 3.5-28.5 years) and 25 BMI- and pubertal stage-matched peers were enrolled in the study. The ISS patients underwent a four-day standard GH stimulation test to evaluate IGF-1 generation. mRNA expression of the IGF-1 R gene in peripheral blood leukocytes was evaluated. ISS patients and controls were compared with respect to anthropometric and laboratory data. The results were also analyzed after subdividing the two groups into low-normal [BMI standard deviation score (SDS) between -2 to -1)] and normal (BMI SDS between -1 to +1) BMI subgroups.

**Results:** Basal serum IGF-1 concentrations were lower in ISS subjects compared to controls who had similar BMI SDS values (p=0.000). Subgroup analyses revealed that there were no significant differences between low-normal BMI ISS subjects and low-normal BMI controls with respect to serum IGF-1 and IGFBP-3 concentrations. However, in the normal BMI ISS subgroup, basal and stimulated IGF-1 levels were significantly lower than the basal values in their control counterparts (basal: p=0.000; stimulated: p=0.007). mRNA expression of IGF-1 R gene was not found to be significantly different in ISS subjects and controls.

**Conclusions:** ISS patients were found to have lower IGF-1 concentrations than BMI-matched peers, a finding supporting presence of an impairment in the somatotropic axis. IGF-1 R expression does not seem to be impaired in ISS patients. ISS patients with low-normal BMI SDS also tend to display a relative IGF-1 resistance, whereas those with normal BMI SDS tend to be less GH-sensitive than healthy peers.

**Conflict of interest:**None declared.

## INTRODUCTION

Idiopathic short stature (ISS) comprises the vast majority of the subjects evaluated for short stature in pediatric endocrine clinics (1).

A growing list of studies continue to highlight the presence of numerous messengers that have functional implications with regard to growth hormone (GH)/insulin-like growth factor 1 (IGF-1) axis including GH receptor, JAK2, STAT5b, STAT5a, MAPK, PI3K, SOCS 1, 2, 3 and several other relevant substances (2). Dysfunction of one or more of these messengers seems to contribute to the clinical scenario of partial GH or IGF-1 resistance or reduced serum IGF-1 concentrations in some subjects rendering a heterogeneous nature to ISS (3,4,5,6,7). The following impairments or a combination of these could operate in subjects with ISS: 1) Heterozygosity of genes in GH/IGF-1 axis resulting in insufficient transcription of IGF-1; 2) Polymorphisms influencing transcription or translation of genes in GH/IGF-1 axis; and 3) Impairment of unidentified genes (8).

Subjects with ISS are reported to have reduced appetite compared to healthy peers, and a lower body mass index (BMI) has been suggested as a contributory factor to short stature. Weight is known to be a regulator of GH/IGF-1 axis (9).

In the present study, we aimed to investigate the differential impact of GH on IGF-1 and IGF-binding protein 3 (IGFBP-3) levels in subjects with ISS and in their healthy peers. GH was administered in a standard-dose (0.033 mg/kg/day) IGF-1 and IGFBP-3 generation test for four days. We also aimed to evaluate the GH/IGF-1 axis in subjects with ISS in relation to BMI and to evaluate whether measuring the expression levels of IGF-1 receptor (IGF-1 R) gene in peripheral blood leukocytes could provide additional insight to the impairment of the growth axis in ISS subjects.

## METHODS

Fifty-five ISS patients (24F/31M) with a mean age of 14.6±4.5 years (range 3.5-28.5 years) followed in our outpatient clinic were included in this case-control study and their medical records were carefully reviewed. All patients were born at term with normal birth weight (BW) and normal birth length (BL). Height standard deviation score (SDS) was estimated using national standards and, at the time of the study, was <-2 SDS in all patients (9). There were no noteworthy laboratory or clinical findings in any of the patients. Some of the patients had reached final height at the time of the study. Normal sitting height/height ratio ruled out skeletal dysplasia as a cause of short stature. Normal biochemical evaluation, thyroid hormone levels, and GH levels on two stimulation tests (peak GH ≥10 ng/mL) excluded chronic diseases, hypothyroidism, and GH deficiency.

Twenty-five (10F/15M) BMI-, pubertal stage- and gender-matched healthy peers with a mean age of 14.1±4 years (range 3.4-26.4 years), with no acute or chronic illness, and with height and weight SDS values within normal ranges ?(>-2 SDS) comprised the controls.

Clinical data of the patients including BW, BL, anthropometric measurements (weight, height, sitting height, BMI) at initial presentation and laboratory data (biochemical analysis, thyroid hormones, and GH stimulation tests) were extracted from the medical records. SDS of BW and BL were calculated using national data (10).

**Anthropometric Data at Current Evaluation**

Height, sitting height and weight measurements on all subjects were done in the morning hours by the same physician (PC) using adjusted, wall-mounted Harpenden height and sitting height stadiometers and an electronic scale sensitive to 100 grams in accordance with standard methods (11). SDS values for anthropometric data and BMI [weight(kg)/height(m)2] were calculated using national data (12,13,14). Pubertal staging of the subjects was done according to the method of Tanner and Marshall (15,16). Height measurements of the parents were done to calculate target height SDS using Tanner’s method (13,17).

ISS patients and controls were divided into two subgroups as those with low-normal and normal BMI values according to their BMI SDS being between -2 SDS and -1 SDS and between -1SDS and +1SDS, respectively (18).

**Laboratory Evaluation**

Venous fasting blood samples were taken in the morning into dry tubes for measurement of serum IGF-1 and IGFBP-3 concentrations and to tubes with EDTA for measurement of mRNA expression levels of IGF-1 R genes. ISS subjects underwent the 4-day standard IGF-1 generation test using recombinant GH (Saizen®, Merck Serono) in a dose of 0.033 mg/kg/day (19). On the 5th day of the test, the blood sampling for measuring the concentration of the same parameters was repeated. Exogenous GH-induced changes in serum concentrations of IGF-1 and IGFBP-3, and the levels of mRNA expression in IGF-1 R were evaluated. SDS values of IGF-1 and IGFBP-3 were calculated using national data (20). Stimulated IGF-1 SDS and IGFBP-3 SDS values were compared with the respective basal values in controls as most of the controls did not give consent to undergo the IGF generation test.

Serum samples for measurement of IGF-1 and IGFBP-3 concentrations were kept at -80°C until analyzed. Serum ?IGF-1 concentrations were measured by immunoradiometric assay (IRMA) (DSL-5600 Active® IGF-1 Coated Tube IRMA kit, DSL, Webster, Texas, USA). Intraassay coefficient of variation (CV) was between 1.5% and 3.4%. Interassay CV ranged between 1.5% to 8.2%. Lowest measurable value of IGF-1 was 0.8 ng/mL. Serum IGFBP-3 concentrations were measured by IRMA (DSL-6600 Active® IGFBP-3 Coated Tube kit, Diagnostic Systems Laboratories, Webster, Texas, USA). Intraassay CV was between 1.8% to 3.9% and interassay CV was between 0.6% to 1.9%. Lowest measurable value for IGFBP-3 was 0.5 ng/mL. Values outside the defined ranges were reevaluated after appropriate dilution of the serum samples.

**Molecular Analyses**

Total RNA was isolated from peripheral blood leukocytes with EDTA obtained from both ISS subjects and controls using MagnaPure RNA isolation kits (Roche, Mannheim, Germany). mRNA expressions of IGF-1 R genes were done by real-time polymerase chain reaction (RT-PCR) using LightCycler 1.5 (Roche Diagnostics, Mannheim, Germany) (20,21). cDNA was prepared by the use of transcriptor first strand cDNA synthesis kit (Roche, Mannheim, Germany). cDNA synthesized from total RNA was used for analyses of the gene expressions. mRNA expression levels of IGF-1 R and Cyclophilin genes were assessed by SYBR green-based detection (Table 1). All samples were amplified in duplicate, and the mean values were obtained for further calculations. The primers were designed using the software Primer 3 v.0.4.0 (http://frodo.wi.mit.edu/primer3/) and synthesized by TIB-MOLBIOL (Berlin-Germany) (21,22). Data to quantify gene expressions were obtained as Ct values (Ct= the cycle number at which logarithmic PCR plots cross a calculated threshold line). Ct values were used to calculate ?CT values (?CT=Ct of the target gene minus Ct of the housekeeping gene). The expression of IGF-1 R gene was compared between depots using the ??Ct method. Statistical analyses of all data were performed at the ?Ct stage in order to exclude potential bias due to averaging of data transformed through the equation 2-??Ct. Data related to quantification of gene expressions were calculated by using the expression of the housekeeping gene (Cyclophilin gene) as an internal standard. The presence of specific gene product was also confirmed with melting curve analysis (22).

## STATISTICAL ANALYSIS

SPSS version 15.0 for Windows and GraphPad Prism 5 were used for the statistical analyses. Student’s t- and paired t-tests were applied in the evaluation of normally distributed variables. Non-parametric tests (Mann-Whitney U, Wilcoxon tests) were used when necessary. Categorical variables were compared using chi-square test and Fisher’s exact test. The relation between variables were analyzed by simple correlation (Pearson’s and Spearman’s tests). Multiple linear regression analysis was used to determine independent factors (BMI SDS, group and basal IGFBP-3) associated with dependent-variable basal IGF-1 SDS. The stimulated concentrations of IGF-1 and IGFBP-3 on four-day standard IGF-1 generation test in ISS subjects were compared to basal IGF-1 and IGFBP-3 concentrations of the controls. Results are expressed as percentages and mean±SD unless stated otherwise. Statistical significance was accepted at a p-value of less than 0.05 and the confidence interval was set at 95%.

**Ethics**

The study was performed in agreement with the Helsinki Declaration and approved by the local ethics committee. Informed consent was obtained from all subjects or from their parents if the subject was under 18 years of age.

## RESULTS

The mean age of the subjects with ISS at initial presentation to our outpatient clinic was 10.7±3.3 years (range 2.3-15.6 years), and the mean duration of follow-up was 3.9±3.0 years (range 1-13.7 years). SDSs of BW and BL were similar in boys (0.2±1.1 and 0.2±0.4, respectively) and girls (-0.4±0.8 and 0±0.7, respectively) and were in normal ranges in all the subjects. Sitting height/height ratios were statistically similar in ISS subjects and controls (0.53±0.01 and 0.53±0.02, respectively; p>0.05), and they were within normal ranges in the whole cohort.

The mean age of the ISS patients and controls at current evaluation was 14.5±4.6 years (range 3.5-28.5 years) and 14.2±4.2 years (range 3.4-26.4 years), respectively (p>0.05). 

**Figure 1:**
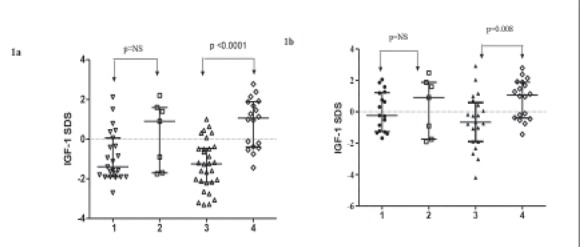
Basal insulin-like growth factor 1 standard deviation score(IGF-1 SDS) levels in low-normal body mass index idiopathic shortstature (BMI ISS) subjects ([ref:1]1[/ref]) and normal BMI ISS subjects ([ref:3]3[/ref]) arecompared to low-normal BMI controls ([ref:2]2[/ref]) and normal BMI controls([ref:4]4[/ref]), respectively (Figure 1a). Stimulated IGF-1 SDS levels in low-normalBMI ISS subjects ([ref:1]1[/ref]) and normal BMI ISS subjects ([ref:3]3[/ref]) are comparedto basal IGF-1 SDS levels of control counterparts (Figure 1b). The horizontallines represent the median with the interquartile ranges

Anthropometric and laboratory data of the whole cohort are shown in Table 2. Height SDS of the ISS group, by definition, was significantly lower than that of the controls. However, BMI SDS values were similar in the groups. Basal IGF-1 SDS (p=0.000) and stimulated IGF-1 SDS (p=0.009) values were significantly lower in ISS patients as compared to the basal IGF-1 SDS values of the controls.Anthropometric and laboratory data of the BMI subgroups of ISS subjects and controls are shown in Table 3. There were no significant differences (except for height SDS by definition) between low-normal BMI ISS patients and low-normal BMI controls with respect to BMI and any laboratory related parameters. Basal IGF-1 SDS was not significantly different in low-normal BMI ISS subjects and their control counterparts (p=0.09). Comparison of normal BMI ISS subjects and BMI-matched controls revealed no difference in anthropometric measurements (except for height SDS by definition). Basal IGF-1 SDS was significantly lower in ISS patients with normal BMI than in BMI-matched controls (p=0.0001). In normal ?BMI ISS patients, both the mean basal and stimulated IGF-1 SDS were significantly lower than the mean basal ?IGF-1 SDS in BMI-matched controls (p<0.001 and p=0.008, respectively) (Table 3) (Figure 1). There were 13 subjects (24%) with familial short stature (FSS) and 42 subjects with non-familial short stature (NFSS) defined according to the classification in the ESPE consensus report (1). The distributions of subjects with FSS and NFSS were comparable between the BMI subgroups of ISS patients (p>0.05) (data not shown). Bone age readings, TH SDS and predicted adult height SDS calculations (when applicable) were comparable between the two subgroups as well (p>0.05) (data not shown).

**Correlation Analyses**

ISS: Basal IGF-1 was significantly correlated with basal IGFBP-3 SDS (r=0.579, p<0.001). Basal and stimulated ?IGF-1 SDSs were not associated with levels of mRNA expression in basal and stimulated IGF-1 R gene. Height SDS and BMI SDS failed to correlate with any of the laboratory related parameters. Correlation analyses yielded similar results in the BMI subgroups of ISS subjects.

Controls: Height SDS and BMI SDS were not significantly correlated with any of the laboratory parameters. Basal IGF-1 was significantly associated with basal IGFBP-3 SDS (r=0.632, p=0.001). Correlation analyses in the BMI subgroups yielded similar findings.

Multivariate linear regression analysis revealed that basal IGF-1 SDS was best explained by (R2=0.52) basal IGFBP-3 SDS (p<0.001) and groups (ISS and controls) (p<0.001). 

## DISCUSSION

The results of this study, conducted on ISS patients and their BMI- and pubertal stage- matched healthy peers, showed the presence of a BMI-independent disturbance in GH/IGF-1 axis in the ISS group. The results of this study also include some findings pointing to an influence of BMI on GH/IGF-1 axis, which probably reflect the complexity of interactions between metabolic signals and the somatotropic axis. The finding of a lower basal and stimulated IGF-1 SDSs in ISS subjects compared to their BMI-matched healthy peers is in favor of a BMI-independent disturbance in IGF axis in ISS subjects. This finding is also in line with other studies (23,24,25,26). However, when we further analyzed the groups with respect to BMI, the difference in IGF-1 concentrations reached significance only in normal BMI ISS patients but not in low-normal BMI ISS subjects, a finding that might indicate a BMI-dependent disturbance in GH/IGF-1 axis in ISS.

The significant correlations of absolute basal and stimulated IGF-1 and IGFBP-3 concentrations in subjects with ISS confirmed the GH responsiveness in our study group - although reduced compared to controls, as described elsewhere (24). In our study, basal and stimulated concentrations of serum IGFBP-3 in ISS patients failed to show any difference from basal IGFBP-3 concentrations in controls, suggesting the unreliability of IGFBP-3 in diagnosing patients with ISS . The reason for the discrepancy in validity of these parameters can probably be attributed to the different cell origins of these molecules - IGF-1 originating from hepatocytes and IGFBP-3 from Kupffer cells (27). One other possibly contributory reason is the presence of a heterogeneous population of subjects with respect to biological maturity, the subjects being children, adolescents, and adults. In order to correct for the difference in biological maturity, we used SDS values of both IGF-1 and IGFBP-3. However, in subjects at puberty, the SDS calculations might yield very negative values, thereby not rendering these calculations totally unbiased (28).

Studies insofar have focused on the tendency of subjects with ISS having lower BMI SDS than healthy peers (9,24). The decreased IGF-1 in ISS subjects was attributed to reduction in non-GH-induced production of IGF-1 as GH-stimulated IGF-1 concentrations were found to be similar in short patients and their healthy peers (29). In our study, we evaluated ISS patients by dividing them into groups according to their BMI SDS values and found that ISS patients with low-normal BMI tended to be relatively IGF-1 resistant, as their basal and stimulated IGF-1 concentrations were statistically similar to the basal IGF-1 concentrations of the controls. Those having normal BMI were found to be more GH-insensitive than controls with normal BMI as their basal and stimulated IGF-1 SDS concentrations were found to be significantly lower than basal serum IGF-1 SDS concentrations in controls, suggesting a different set point of IGF-1 regulation in ISS patients with normal BMI. Furthermore, the standard IGF-1 generation test yielded lower mean IGF-1 concentrations in normal BMI ISS subjects than the basal IGF-1 concentrations in the low-normal BMI ISS subgroup. A study suggesting the heterogeneous nature of subjects with ISS having relative IGF-1 or GH insensitivity has recently been published (4). In our study, we observed that changes in BMI within normal ranges rendered the ISS partially IGF-1- or GH-insensitive. However, this was a weak observation given in the context of a relatively low number of controls in the low-normal subgroup.

Abnormalities of GH postreceptor signaling and IGF-1 R have been described in some ISS patients (30,31). In our study, evaluation of IGF-1 R gene expression in peripheral blood leukocytes did not reveal any significant difference in low-normal BMI ISS patients and controls suggesting a postreceptor defect in IGF-1 signaling in ISS subjects with low-normal BMI. The presence of partially IGF-1 resistant, partially GH-insensitive subjects in ISS groups in our cohort suggests presence of subtle defects in signaling with some contribution from change in BMI SDS. Indeed, a recent study revealed the presence of heterozygous STAT5B mutations in some ISS subjects with GH insensitivity (32).

We acknowledge the fact that serum IGF-1 concentrations do not entirely account for the growth process and that GH itself has direct influence on growth (24). We also acknowledge that short subjects tend to have subnormal growth even if similar IGF-1 values are attained compared to those of healthy peers (4). Serum IGF-1 concentrations are known to reflect tissue levels to some extent and they are used as surrogate markers of growth and therapeutic adequacy in subjects receiving GH therapy. Regarding the protocol of IGF generation test, the test protocol we used in our study is currently the most commonly used one worldwide (19). However, different protocols have been used and even an acute response to a single dose of GH has been evaluated (33). It can be speculated that nutrition may need to be controlled for in low BMI ISS subjects. However, to avoid bias, we made the inferences of our data with respect to BMI-matched controls and all the subjects in our cohort had BMI SDS >-2 SDS.

One limitation of our study is that the number of subjects in the low-normal BMI control subgroup is relatively low. However, even if the control group is not taken into account, the significant difference in the stimulated IGF-1 levels on IGF generation test between the low-normal BMI ISS subgroup and normal BMI ISS subgroup is indicative of a different abnormality in GH/IGF-1 axis in ISS subjects with different BMI values.

In conclusion, our study suggests that, owing to their reduced IGF-1 SDSs and independent of BMI, a different set point of IGF-1 regulation is present in ISS patients. Yet, the change in BMI values within normal ranges tends to change the set point. ISS patients with low-normal BMI might have a relative IGF-1 resistance, whereas those with normal BMI tend to be less GH-sensitive, which might suggest the need for different therapeutic strategies in these children. However, this finding awaits confirmation owing to the limitations mentioned above. Evaluation of gene expressions of IGF-1 R failed to provide an insight to the pathogenesis of ISS.

## Figures and Tables

**Table 1:**
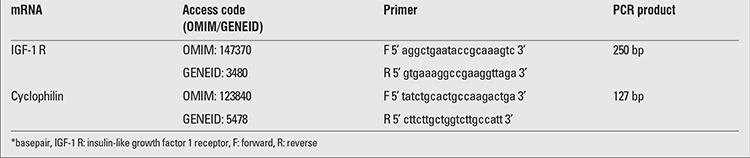
Nucleotide sequences of primers and the size of polymerase chain reaction (PCR) products in the study

**Table 2:**
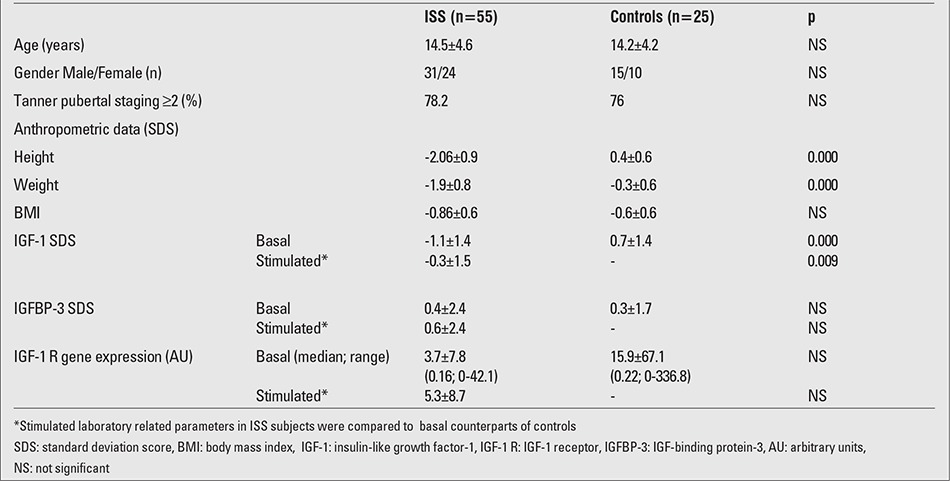
Anthropometric and laboratory data of idiopathic short stature (ISS) patients and the control group

**Table 3:**
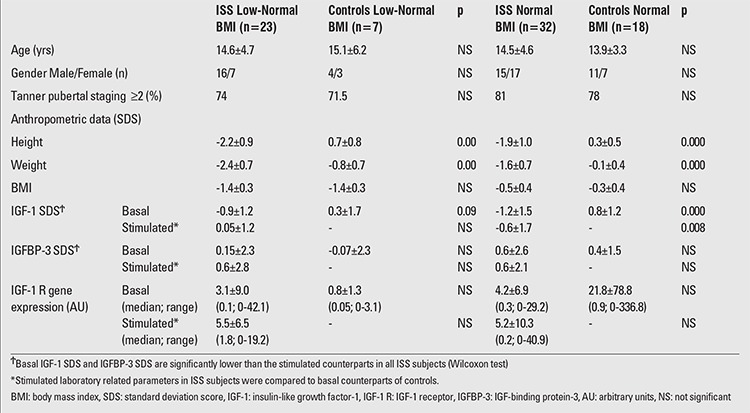
Anthropometric and laboratory data of idiopathic short stature (ISS) patients and controls in the body mass index (BMI) subgroups
